# The interaction between *Staphylococcus aureus* SdrD and desmoglein 1 is important for adhesion to host cells

**DOI:** 10.1038/srep22134

**Published:** 2016-02-29

**Authors:** Fatemeh Askarian, Clement Ajayi, Anne-Merethe Hanssen, Nina M. van Sorge, Ingvild Pettersen, Dzung B. Diep, Johanna U. E. Sollid, Mona Johannessen

**Affiliations:** 1 Research group of Host-Microbe Interactions, Department of Medical Biology, Faculty of Health Sciences, UiT-The Artic University of Norway, Norway; 2 Medical Microbiology, University Medical Center Utrecht, Utrecht 3584CX, The Netherlands; 3 Department of Chemistry, Biotechnology and Food Science, Norwegian University of Life Science, Ås, Norway

## Abstract

*Staphylococcus aureus* is known as a frequent colonizer of the skin and mucosa. Among bacterial factors involved in colonization are adhesins such as the microbial surface components recognizing adhesive matrix molecules (MSCRAMMs). Serine aspartate repeat containing protein D (SdrD) is involved in adhesion to human squamous cells isolated from the nose. Here, we identify Desmoglein 1 (Dsg1) as a novel interaction partner for SdrD. Genetic deletion of *sdrD* in *S. aureus* NCTC8325-4 through allelic replacement resulted in decreased bacterial adherence to Dsg1- expressing HaCaT cells *in vitro*. Complementary gain-of-function was demonstrated by heterologous expression of SdrD in *Lactococcus lactis*, which increased adherence to HaCaT cells. Also ectopic expression of Dsg1 in HEK293 cells resulted in increased adherence of *S. aureus* NCTC8325-4 *in vitro*. Increased adherence of NCTC8325-4, compared to NCTC8325-4Δ*sdrD,* to the recombinant immobilized Dsg1 demonstrated direct interaction between SdrD and Dsg1. Specificity of SdrD interaction with Dsg1 was further verified using flow cytometry and confirmed binding of recombinant SdrD to HaCaT cells expressing Dsg1 on their surface. These data demonstrate that Dsg1 is a host ligand for SdrD.

*Staphylococcus aureus* is a human commensal that frequently colonizes the human skin and mucosa, either for long or short periods throughout life^1^,^2^. It is also an important cause of several life-threatening infections. The ability of *S. aureus* to efficiently colonize the host epithelium, invade tissues and survive within host cells is regulated through numerous adhesive and invasive factors^3^,^4^.

*S. aureus* expresses a panel of cell-wall anchored adhesins including the microbial surface components recognizing adhesive matrix molecule (MSCRAMM) families, which target extracellular matrix proteins and other molecules on host cells^5^,^6^,^7^,^8^. The Clumping factor (Clf) and Serine aspartate repeat containing protein (Sdr) families of MSCRAMMs share structural features. They contain N-terminal signal peptide followed by an A region (divided into distinct sub-domains called N1, N2 and N3), two to five B repeats, an R domain (Ser-Asp repeats), a LPXTG cell wall-anchoring motif, a hydrophobic membrane spanning region, and a cytoplasmic C-terminal end^8^,^9^ (Fig. 1a).

Bacteria can target intercellular junctions on host cells to promote adhesion and/or internalization during colonization and infection^10^,^11^,^12^. Desmosomes are the main type of adhesive disk shaped intercellular junctions, abundant in tissues exposed to mechanical stress, such as skin and heart^13^. The desmosomal cadherins, desmogleins (Dsgs) consist of extracellular, transmembrane and cytoplasmic domains^14^. The extracellular domain is calcium-dependent and composed of 4 cadherin repeats (EC 1–4), and the extracellular anchor domain (EA), while the intracellular tail binds to adaptors connected to the intermediate filaments of host cytoskeleton^15^. The expression pattern of desmoglein isoforms 1–4 in human epidermis is highly variable^16^,^17^,^18^. These expression patterns impact on the barrier function^19^ and also the differentiation of the epithelial tissues^20^.

Dsg1 is expressed throughout all the nucleated cell layers of human epidermis^18^ and recent evidence suggests that either Dsg1 or Dsg3 can compensate for the adhesive functions of each other in different epithelia tissues^21^,^22^. Dsg1 has been shown to mediate keratinocyte differentiation in the epidermis^23^. In addition, Dsg1 plays a key role in the pathogenesis of three different dermatological conditions including; pemphigus foliaceus, staphylococcal scalded skin syndrome and striate palmoplantar keratoderma (reviewed in^24^). It has also been demonstrated that mutations in the *dsg1* gene results in severe dermatitis, multiple allergies and metabolic wasting^25^.

The role of MSCRAMMs in *S. aureus* colonization and infection has been demonstrated previously (reviewed in^8^). Several of the MSCRAMMs are involved in attachment of *S. aureus* to squamous epithelial cells^26^,^27^,^28^ and keratinocytes^29^ *in vitro* as well as promoting nasal colonization in mice^27^,^30^ and humans^31^. SdrD specifically promotes adherence of bacteria to desquamated nasal epithelial cells, harvested from human donors^26^. Thus, we hypothesize that SdrD may promote colonization through interaction with particular host molecules. The aim of this study was to identify a host ligand for *S. aureus* SdrD and to investigate the potential effect of this interaction on *S. aureus* colonization of host cells.

## Results

### *sdrD* gene localization and expression in *S. aureus* NCTC8325-4

**Figure 1:**
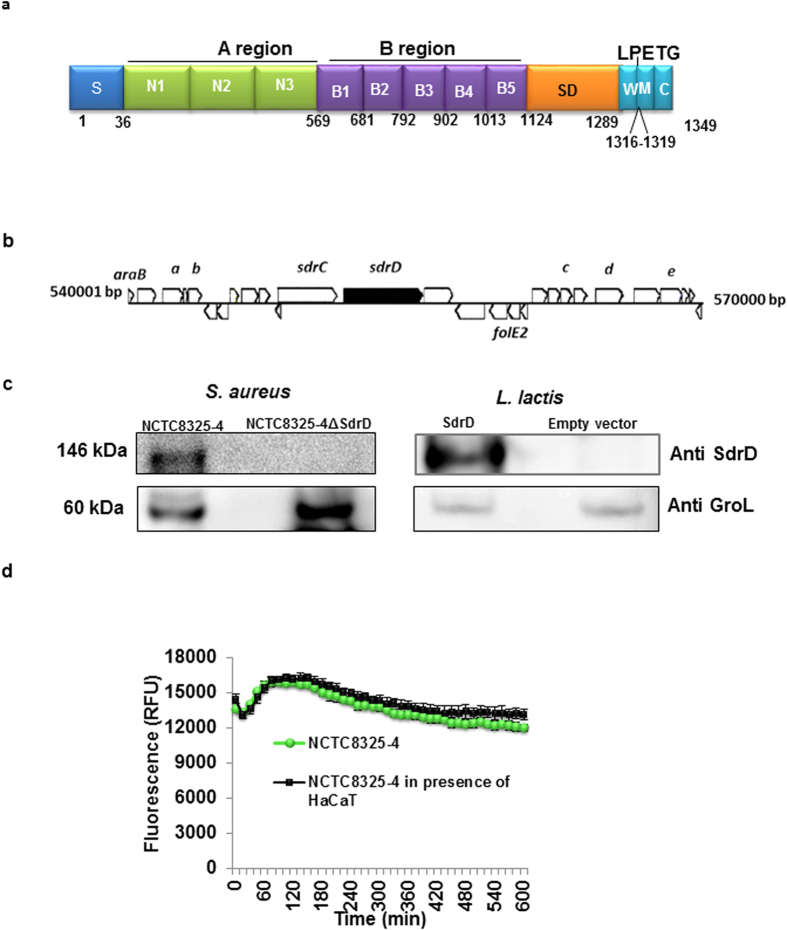
s*drD* gene localization and expression in *S. aureus* NCTC8325-4. (**a**) Schematic representation of SdrD domain structure in *S. aureus* NCTC8325-4 based on UniProtKB. S, signal sequence; A region composed of N1, N2 and N3; B repeats composed of B1 to B5; SD, serine-aspartate acid repeat region; W, wall-spanning fragment; LPETG, cell wall anchoring motif; M, transmembrane domain; C, cytoplasmic domain. (**b**) *sdrC* and *sdrD* is located between ORFs encoding hypothetical proteins according to annotation is from KEGG Genome map. Gene and protein name based on UniProtKB: *araB,* ribonuclokinase; *a*: SAOUHSC_00536, Branched-chain-amino-acid aminotransferase; *b*: SAOUHSC_00538, Haloacid dehalogenase-like hydrolase; *sdrC*, serine-aspartate repeat-containing protein C; *sdrD*, serine-aspartate repeat-containing protein D; *folE2,* GTP cyclohydrolase FolE2; *c*: SAOUHSC_00554, SIS domain protein; *d*: SAOUHSC_00556, Proline/betaine transporter; *e*: SAOUHSC_00558, Acetyl-CoA acetyltransferase (**c**) Immunoblot using SdrD A-region and GroL antibodies on cell lysate *S. aureus* NCTC8325-4 or its isogenic mutant NCTC8325-4Δ*sdrD* and *L.lactis* with pMG36e-SdrD (SdrD) or pMG36e (empty vector). (**d**) *sdrD* promoter activity in DMEM supplemented with FBS without agitation in the absence (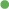) or presence (■) of HaCaT cells using *S. aureus* NCTC8325-4 harbouring *sdrD*-GFP reporter construct. Data expressed as mean ± standard deviation (SD) of an individual experiment.

The *sdrD* gene encodes LPXTG-anchored protein of 1349 amino acids that is composed of an anterior A region (residues 36–568), a medial B region (residues 569–1123) and a posterior SD repeat R region (residues 1124–1289) (Fig. 1a). The *sdr* locus of *S. aureus* consists of *sdrC, sdrD* and/or *sdrE*, but may not all be present in the same strain^6^,^7^. From the annotation, the *sdr* locus of NCTC8325-4 contains *sdrC* and *sdrD* (Fig. 1b).

By allelic replacement, a NCTC8325-4Δ*sdrD* mutant was created. Bacterial growth was not significantly affected by deletion of *sdrD* (*P* < 0.05). Furthermore, both strains were haemolytic as visible on the blood agar plates (see supplementary Fig. S1). A heterologous expression system was constructed by expressing SdrD in the non-invasive bacterium *L.lactis*. Immunoblot analysis showed that the expression of SdrD was detected when *sdrD* gene was present in *S. aureus* and *L.lactis* (Fig. 1c, upper lane). The presence of bacterial lysate was proven by immunoblot of GroL (Fig. 1c, lower lane).

The expression pattern of *sdrD* in NCTC8325-4 was assayed in eukaryotic cell culture medium (DMEM supplemented with FBS) in the absence or presence of HaCaT cells by use of a *sdrD*-specific GFP reporter construct. Immediately after inoculation, the expression of GFP showed a slight decrease, followed by an increase (Fig. 1d). The expression of the reporter was similar in the presence or absence HaCaT cells. Expression of SdrD in presence of HaCaT was detected in NCTC8325-4, but not in its isogenic mutant as confirmed by immunoblot (data not shown).

### The presence of SdrD promotes adhesion of *S. aureus* in HaCaT cells

**Figure 2:**
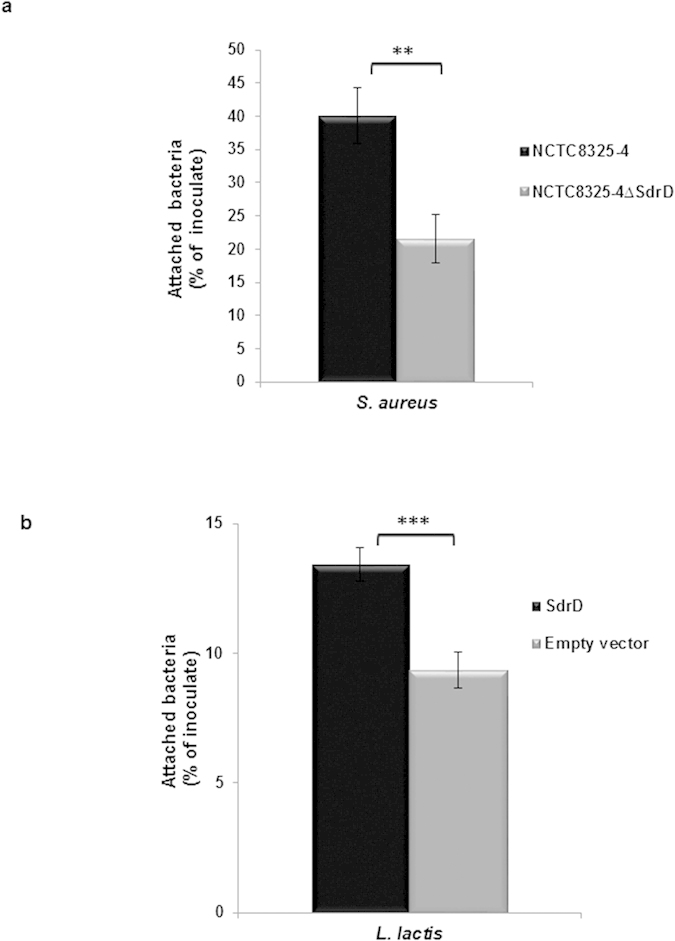
SdrD mediates adherence of *S. aureus* and *L. lactis* to HaCaT cells. Adherence of (**a**) *S. aureus* NCTC8325-4 and its isogenic mutant NCTC8325-4Δ*sdrD* and (**b**) *L. lactis* with pMG36e-*sdrD* (SdrD) or pMG36e (empty vector) to HaCaT cells. The number of inoculated bacteria was arbitrarily set as 100% and the number of attached bacteria represented as the mean percentage of inoculate. Data represent means ± SEM of 4 independent experiments. Statistical analysis was performed by Student’s t-test. Significant differences are indicated by two (*P* < 0.01), or three (*P* < 0.001) asterisks (*).

To investigate the contribution of SdrD in adherence to human keratinocytes, *S. aureus* NCTC8325-4 and the isogenic mutant NCTC8325-4Δ*sdrD* were incubated with confluent layers of HaCaT cells. Unbound bacteria were removed by washing and adherent bacteria were quantified by plating serial dilutions. The presence of SdrD promoted better adherence of NCTC8325-4 to HaCaT cells, as the isogenic mutant showed two-fold reduction in adherence (*P* < 0.05) (Fig. 2a). *S. aureus* internalization into HaCaT cells has been demonstrated previously^32^,^33^. However, the internalized bacteria did not exceed 0.8% of the adhered bacteria (results not shown).

*S. aureus* expresses MSCRAMMs which might cause some functional redundancy in adherence assays^8^. Therefore, *sdrD* was cloned into a lactococcal vector (pMG36e) allowing heterologous expression in *L. lactis*. In adhesion assay performed using *L. lactis*, a significant increase was observed when *L. lactis* expressed SdrD compared to *L. lactis* carrying only empty vector (Fig. 2b). Through this approach, the redundancy challenges associated with *S. aureus* adhesins is avoided, and the effect of SdrD on bacterial adhesion could be observed. These results confirm that SdrD contributes to the *S. aureus* adhesion to HaCaT cells.

### The presence of SdrD promotes *S. aureus* attachment to human desmoglein 1

To identify the host interaction partner within human skin, a yeast-two hybrid screen was performed using GAL4-SdrD A-region as bait in human reconstituted skin library. By this approach, desmoglein 1 (Dsg1) was identified as a putative interaction partner.

**Figure 3:**
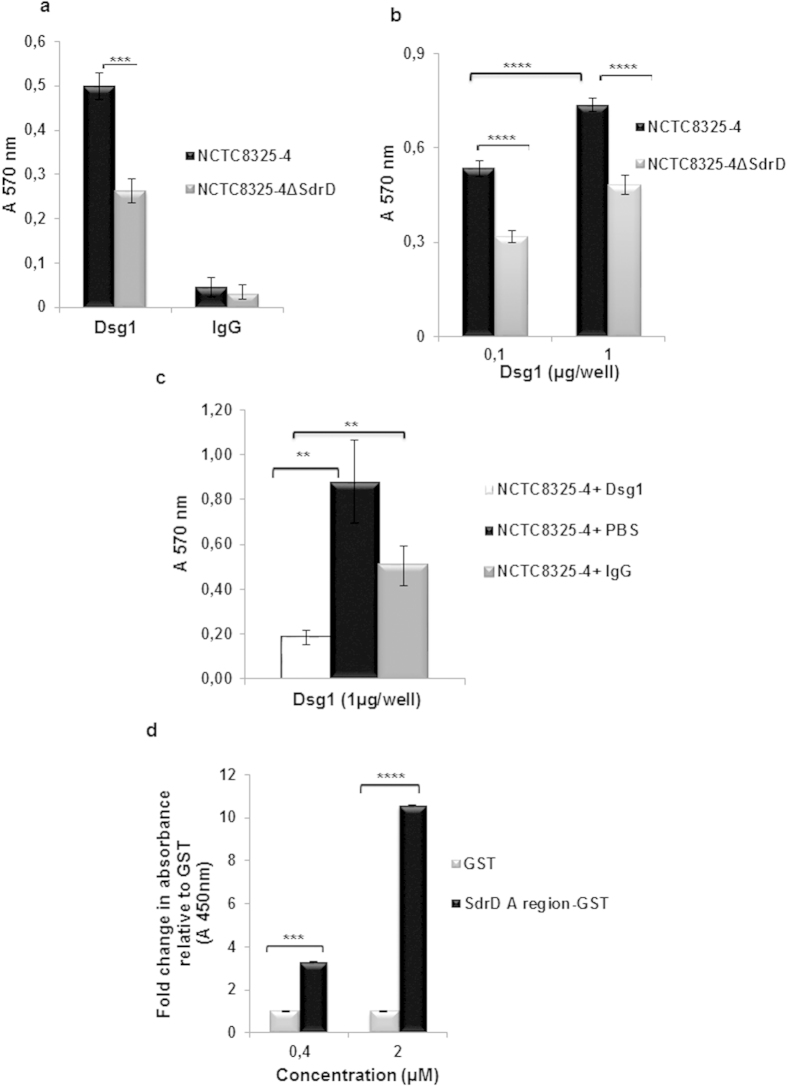
Dsg1 is a host ligand for SdrD. (**a**) *S. aureus* NCTC8325-4 and the isogenic mutant NCTC8325-4Δ*sdrD* were added to wells coated with Dsg1-Fc chimera or control IgG_1_ Fc. Bacterial adherence was measured by staining with crystal violet and measurement of the absorbance at 570 nm. (**b**) Binding of *S. aureus* to Dsg1 is concentration dependent. Different concentrations of Dsg1 were coated on ELISA plates and bacterial adherence was evaluated as described in a. (**c**) *S. aureus* NCTC8325-4 was pre-incubated with IgG_1_ Fc or Dsg1-Fc chimera and added to Dsg1-coated ELISA wells (1 μg/well). Bacterial adherence was evaluated as described in legend a. (**d**) Wells were coated with GST-SdrD-A region and GST before adding of Dsg1. Adherence of Dsg1 was determined using Dsg1-specific antibodies, followed by HRP-conjugated secondary antibody. The measurement of absorbance at 450 nm was carried out using an ELISA reader (VERSA_max_, USA). The absorbance value for GST was arbitrarily 1 and the absorbance in presence of SdrD-A region is represented as fold change. Data represent means ± SEM of at least 3 independent experiments. Statistical analysis was performed by Student’s t-test. Significant differences are indicated by ns (no statistical significance), two (*P* < 0.01), three (*P* < 0.001) or four (*P* < 0.0001) asterisks (*).

To assess Dsg1 as binding partner, we first studied the ability of SdrD to facilitate the adherence of *S. aureus* to Dsg1-coated microtiter plates. Wells were coated with Dsg1 Fc-chimera, IgG_1_ Fc or buffer only and the adherence of *S. aureus* NCTC8325-4 and its isogenic SdrD mutant were compared. *S. aureus* NCTC8325-4 adhered significantly better to immobilized Dsg1 than NCTC8325-4Δ*sdrD* (*p* < 0.05) (Fig. 3a). Similarly, when the amount of Dsg1 was raised from 0.1 to 1 μg/well, the bacterial adhesion was increased (Fig. 3b).

If bacteria expressing SdrD bind Dsg1 in solution, one would expect that the bacterium is coated with the protein and bind less efficiently to the immobilized Dsg1. Competition-based studies were performed where *S. aureus* NCTC8325-4 was pre-incubated with Dsg1, PBS or IgG1 Fc (the two latter as negative controls) before being allowed to adhere to immobilized Dsg1. As shown in Fig. 3c, pre-incubation of *S. aureus* NCTC8325-4 with Dsg1 considerably reduced its adhesion ability to immobilized Dsg1. This reduction in adherence was not seen or was much less when cells were pre-incubated with PBS or IgG1 Fc, respectively, thus showing that the binding between SdrD and Dsg1 is specific.

Finally, the interaction was evaluated using purified proteins. Thus, the microtiter plate was coated with two concentrations of immobilized GST-SdrD (A-region), prior to addition of recombinant Dsg1-protein. The binding of Dsg1 was thereafter evaluated using Dsg1-specific antibodies. As seen in Fig. 3d, recombinant Dsg-1 bound in a dose-dependent manner to immobilized SdrD (A region) protein. There was no significant binding between GST and Dsg1. All these results suggest a direct interaction between SdrD and Dsg1 *in vitro*.

### Recombinant SdrD binds to HaCaT cells expressing Dsg1 and ectopic Dsg1 expression promotes SdrD-mediated *S. aureus* adhesion to HEK293 cells

**Figure 4:**
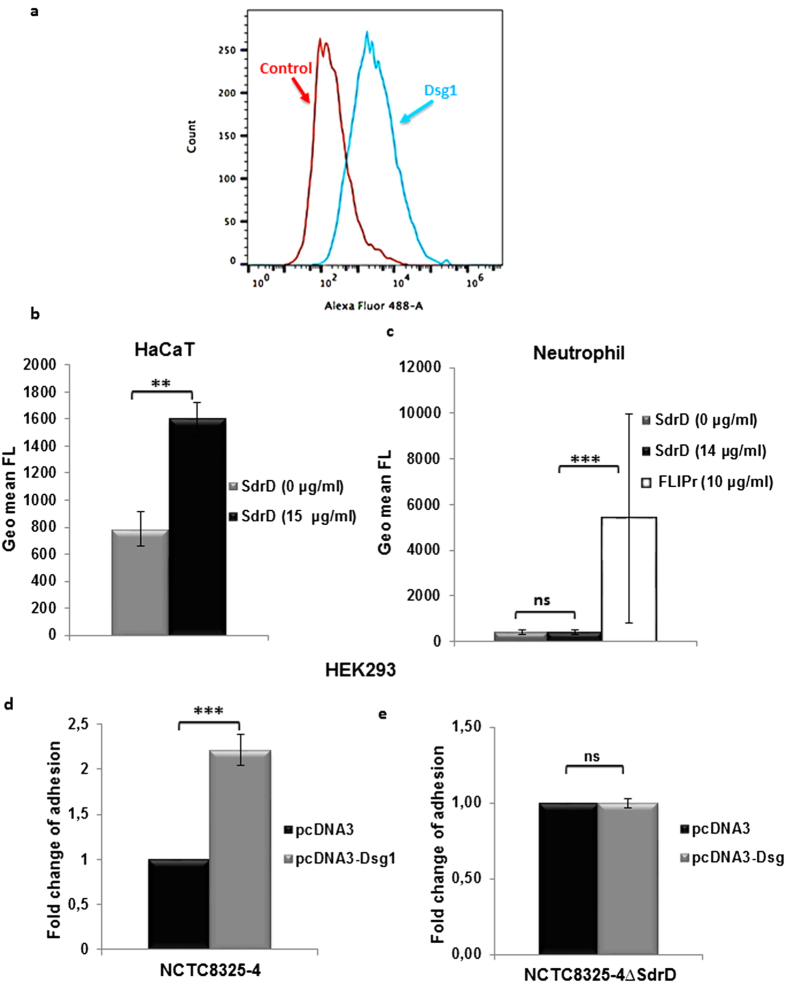
SdrD specifically binds and promotes *S. aureus* adherence to the host cells expressing Dsg1. (**a**) Dsg1 is expressed surface of HaCaT cells as measured by flow cytometry (blue histogram). Control antibody binding to cells in red histogram. (**b**) Binding of his-tagged SdrD (full length) to HaCaT cells as detected with anti-his-FITC by flow cytometry. The results are presented as geometric mean of the fluorescence intensity (FL). (**c**) Binding of his-tagged SdrD (full length) or FLIPr (positive control) to neutrophils measured as indicated in the figure legend b. (**d**) Ectopic expression of Dsg1 in HEK293 promotes adherence of NCTC8325-4 but (**e**) not NCTC8325-4Δ*sdrD*. The cells transfected with empty vector were arbitrarily set as 1, and the fold change of adherence in pcDNA3-Dsg1 transfected is represented as fold change. Data represent means ± SEM of at least 3 independent experiments. Statistical analysis was performed by Student’s t-test. Significant differences are indicated by ns (no statistical significance), two (*P* < 0.01), or three (*P* < 0.001) asterisks (*).

HaCaT cells express Dsg1, which is predominantly located in the upper layer of epidermis^34^. Expression of Dsg1 on the surface was confirmed by flow cytometry using Dsg1-specific antibody followed by detection with an Alexa-488 conjugated secondary antibody (Fig. 4a). To verify specificity of SdrD for Dsg1, recombinant full-length SdrD (see supplementary Fig. S2) were added to HaCaT cells and neutrophils. As shown in Fig. 4b, full length SdrD significantly bound to HaCaT cells expressing Dsg1 on their surface but not to neutrophils (Fig. 4c), which do not express Dsg1^23^,^35^. On the other hand, *S. aureus* formyl peptide receptor inhibitor (FLIPr) bound neutrophils as expected^36^. Thus, SdrD specifically targets host cells expressing Dsg1.

Next, we assessed whether ectopic Dsg1 expression can promote *S. aureus* NCTC8325-4 adhesion to host cells. HEK293 cells were transfected with pcDNA3-Dsg1 (encoding Dsg1) or the control plasmid pcDNA3 and their binding to NCTC8325-4 or the isogenic NCTC8325-4Δ*sdrD* (negative control) was assessed. The number of adhered *S. aureus* NCTC8325-4 was significantly increased in HEK293 cells transfected with pcDNA3-Dsg1 compared to HEK293 cells with the control plasmid pcDNA3 (Fig. 4d). Such an increase was not seen when assayed with the isogenic mutant (Fig. 4e). A direct comparison, using a five-fold increased MOI, confirmed that Dsg1 contributes in SdrD-mediated *S. aureus* attachment to the host cells (Supplementary Fig. S3).

Taken together, these results confirm that SdrD contributes significantly to adherence of *S. aureus* to host cells using Dsg1 as its interaction partner.

## Discussion

Host adhesion molecules such as cadherins, integrins or immunoglobulin-related cell adhesion molecules (ICAMs) are connected to the host cytoskeleton, and are often used as targets for bacterial adhesion as well as internalization^12^. *S. aureus* binds specifically integrins through a fibronectin-bridge, while *Haemophilus influenzae* is an example of a bacterium that binds ICAMs. The EC1 domain of E-cadherin is a ligand for *Listeria* InlA^12^, while desmosomal cadherin Dsg2 is the receptor for Adenovirus^37^. In this study, we demonstrate for the first time that *S. aureus* SdrD interacts directly with Dsg1. The interaction was confirmed using several approaches including yeast two hybrid (Hybrigenics) and ELISA based assays using purified recombinant SdrD and Dsg1 proteins. Additionally, disruption of *sdrD* in *S. aureus* NCTC8325-4 attenuated adhesion to immobilized Dsg1 and to human keratinocytes. However, there is a high functional redundancy among MSCRAMMs^8^. Since interaction with keratinocytes is not completely lost by deletion of SdrD, our data suggest that Dsg1 may also interact with other *S. aureus* adhesion molecules (Fig. 3a,b). Complementary, ectopic surface expression of Dsg1 in HEK293 cells significantly increased adhesion of SdrD-expressing *S. aureus* and purified full-length SdrD protein binds to HaCaT cells that expressed Dsg1. All these results provide evidence for a direct interaction between SdrD and Dsg1, which improves bacterial adherence to keratinocytes.

Colonization is a well-known risk factor for development of staphylococcal infections, and adherence of *S. aureus* to the host cells promotes colonization^38^. The molecular aspects of colonization include interaction between bacterial adhesins and host molecules. Clumping factor B has recently been found to support colonization through its binding to loricrin^39^. Loricrin is highly expressed in stratum corneum^40^, and the interaction between loricrin and ClfB may be especially important in the initial phase of colonization. ClfB has previously also been found to interact with keratin 8 and 10 (reviewed in^8^), and cytokeratin 10 is expressed in the suprabasal layer of epidermis^41^. Surprisingly, bacteria are present in all layers of human epidermis and even sub-epidermal compartment of human skin^42^, Here we have established that the SdrD–Dsg1 interaction promotes adhesion to keratinocytes. The desmosomal structure of which Dsg1 is part of, is connected to the keratins^18^, and it is tempting to speculate that both SdrD and ClfB may be of importance later during the colonization throught their interaction with Dsg1 and cytokeratin 10, respectively. However, although SdrD is shown to contribute to bacterial adhesion to human squamous cells isolated from the nose^26^, and its expression increases during nasal colonization^43^, it is not known whether SdrD-Dsg1 interaction promotes nasal colonization.

Keratinocytes constitute a large part of the epidermis^44^, and Dsg1 contributes to maintaining the structural epidermal integrity in stratified epidermal cells^45^. Dsg1 is targeted by *S. aureus* exfoliative toxin, resulting in loss of cell-cell adhesion in the epidermis^46^. Moreover, in the immune disease pemphigus, the tissue integrity is disrupted by autoantibodies against Dsg1/3^47^. One hypothesis is that SdrD-Dsg1 interaction may also affect the structural integrity of the epidermis. However, subsequent molecular mechanism of this interaction remains to be investigated.

In addition, Dsg1 influences keratinocyte differentiation via its influence on the Ras intracellular signalling^25^. Thus, another hypothesis is that SdrD-Dsg1 interaction influences the ability of keratinocytes to effectively differentiate into respective cellular structures within the epidermal-stratified structure. Dsg1 also influences the Rho signalling pathway^48^, an essential pathway affecting cytoskeleton and overall migration of cells^49^. This scenario could possibly influence the ability of *S. aureus* to invade deep tissues and subsequently enter into the bloodstream. SdrD expression is regulated in the presence of whole human blood^50^. Thus this interaction might be essential for the invasion and subsequent migration of the bacterium from the site of colonization.

In conclusion, we have identified Dsg1 as a novel host ligand for *S. aureus* SdrD and demonstrated the importance of this interaction in the promotion of *S.aureus* adhesion to host cells. This interaction may initiate further biological processes, which could influence the pathogenesis and progression of *S. aureus* infections. A deeper understanding of the molecular mechanism of this interaction may aid in the development of anti-virulence strategies/therapeutics to combat *S. aureus* colonization of the epidermis.

## Materials and Methods

### Bacterial strains, mammalian cell lines and growth conditions

*Staphylococcus aureus* subsp. *aureus* NCTC8325-4 was used for allelic replacement and cloning of *srdD* for expression constructs. *Lactococcus lactis* MG1363 was used for heterologous expression assays^51^. *S. aureus* was cultured in tryptic soy broth (TSB) at 37 °C with shaking at 220 rpm. *L. lactis* MG1363 was cultured in SMG17 (M17 broth supplemented with 0.5M sucrose (Sigma Aldrich, Germany) and 0.5% glucose (Sigma Aldrich, Germany) at 30 °C, without shaking. For adherence assays, overnight bacterial cultures were diluted 1:100 in appropriate culture medium and grown to OD_600nm_ = 0.7–0.8 for *S. aureus*, and to OD_600nm_ = 0.5–0.6 for *L. lactis*, before the bacteria were pelleted, washed twice in 1 × PBS (37 °C) and in DMEM media supplemented with 10% FBS.

HaCaT cells, a human keratinocyte cell line^52^ and HEK293 cells, a human embryonic kidney cell line, were purchased from PromoCell (Germany) and European collection of Cell Cultures (Porton Down, UK), respectively. The cells were maintained in Dulbecco’s modified Eagle’s medium (DMEM) (Sigma Aldrich, Germany), supplemented with 10% (v/v) fetal bovine serum (FBS) (Invitrogen Life Technologies, USA), penicillin (100 units/ml), and streptomycin 100 μg/ml (Sigma Aldrich, Germany) in a CO_2_ incubator (5% CO_2_) at 37 °C.

Neutrophils were freshly isolated from heparinized venous blood of healthy volunteers (see section for ethical approval) using Histopaque (Sigma Aldrich, Germany)-Ficoll-paque (GE Healthcare, Sweden) gradient centrifugation. Neutrophils were kept in RPMI 1640 (Gibco, Life Technologies, UK), supplemented with 0.05% human serum albumin (HSA) (Sanquin, Amsterdam, The Netherlands).

### Genetic manipulation of *S. aureus*

Markerless precise allelic replacement of *sdrD* was performed in *S. aureus* NCTC8325-4 using previously described methods^53^ with minor modifications^54^. Briefly, DNA fragments 1050 bp upstream and 1025 bp downstream of *sdrD* were amplified using primers Up For *sdrD* + attB1, Up Rev *sdrD*, Down For *sdrD* and Down Rev *sdrD* + attB2 (Table 1). The upstream and downstream PCR products were fused by PCR using Up For *sdrD* + attB1 and Down Rev *sdrD* + attB2 primers. The fusion construct was subcloned into a temperature sensitive plasmid, pKOR1^55^, using Gateway BP clonase II enzyme mix (Invitrogen, USA) and transformed into *S. aureus* NCTC8325-4 through electroporation. A temperature shifting and antisense counter selection resulted in precise allelic replacement of *sdrD*. Deletion of *sdrD* was confirmed by PCR using primers *sdrD* KO confirm For + *sdrD* KO confirm Rev and *sdrD* Int For + *sdrD* Int Rev (Table 1), and by Western blot.

### Heterologous expression of SdrD in *L. lactis*

For heterologous expression of SdrD in *L. lactis* MG1363, *sdrD* open reading frame from NCTC8325-4 genomic DNA was amplified using primers *sdrD-* pMG36e For and *sdrD-* pMG36e Rev (Table 1). The PCR product was digested using *Xba*I and *Xho*I, and ligated to the corresponding sites of pMG36e^56^, yielding pMG36e-SdrD. pMG36e control or pMG36e-SdrD constructs were transformed into *L. lactis* M1363 through electroporation (100-Ω resistance, 25 μF capacitance and 2.5 kV voltage). Transformants were selected and maintained in M17 medium (Oxoid, UK) containing 0.5% glucose and 10 μg/ml erythromycin. The expression of SdrD in the transformed *L.lactis* M1363 was evaluated by Western blot using antibodies against the SdrD-A region (kind gift from Dr. Elisabet Josefsson).

### Expression and purification of recombinant SdrD

Prokaryotic expression constructs for recombinant SdrD were created as follows: The *sdrD* gene of *S.aureus* NCTC 8325-4 was amplified by PCR using either SdrD- Full length-F and SdrD- Full length-R or SdrD-A-F and SdrD-A-R (Table 1) primers. The PCR products with full-length or the A-region of SdrD were ligated to the corresponding sites of pRSETB vector (Invitrogen) or pGEX4T-1 (GE healthcare), respectively. Both plasmids were expressed in *E. coli* BL21 (DE3) and BL21, respectively. Briefly, recombinant full-length SdrD was expressed upon overnight incubation at 20 °C using Luria broth (LB) media supplemented with ampicillin and glucose. IPTG was added to a final concentration of 1mM, and the induction occurred overnight. Recombinant full-length SdrD was purified from pRSETB-SdrD by Norwegian Structural Biology Center (http://norstruct.uit.no) using His Trap column. According to the company, the purity of the full-length His-tagged SdrD protein is 85%. A selected fraction (supplementary Fig. S2, marked with red) was sent for mass spectrometry analysis and SdrD (gi 44685714) was detected as one of the protein hits (http://www.matrixscience.com/cgi/master_results.pl?file=..%2Fdata%2F20141209%2FFTgmOeamL.dat). GST-SdrD (A-region) was purified from pGEX4T-1-SdrD-A according to the manufacturer’s instructions.

### Immunoblot

Immunoblot analysis was carried out on bacterial lysates. Cells of *S. aureus* and *L. lactis* were pelleted, dissolved in B-PER (Thermo Scientific, UK) and treated with 20 μg/ml lysostaphin (Sigma Aldrich, Germany) for 30 min at room temperature and 10 mg/ml lysozyme (Sigma Aldrich, Germany) for 1 hour at 37 °C, respectively. Bacterial lysates were freeze-thawed three times, followed by sonication. Aliquots of the bacterial lysates were used for western blot. The expression of SdrD was evaluated by immunoblot using antibodies against the SdrD-A region (kind gift from Dr. Elisabet Josefsson) as primary antibody and polyclonal Swine Anti Rabbit immunoglobulin (DAKO, Denmark) as secondary antibody. The GroL antibody (kind gift from Nikola Zlatkov Kolev) was used as control.

### *sdrD* expression in the absence and presence of eukaryotic cells

An *sdrD*-GFP -reporter construct was generated by amplifying the *sdrD* promoter region by PCR from genomic DNA of *S. aureus* NCTC8325-4 using primers *sdrD* prom For and *sdrD* prom Rev (Table 1). The PCR product was ligated into the corresponding sites of pCM29^57^ upstream of GFP and transformed into *S. aureus* NCTC8325-4 by electroporation as previously described^54^. Expression of *sdrD-*GFP in *S. aureus* NCTC8325-4 was assessed in the absence or presence of HaCaT cells in DMEM supplemented with 10% FBS (without agitation). HaCaT cells were seeded at a density of 2 × 10^4^ cells/well into 96 wells microtiter plate (Corning, USA) in DMEM 10% FBS and infected with *S. aureus sdrD*-GFP reporter strain. For this purpose, an overnight culture of *S. aureus* NCTC8325-4 harbouring the *sdrD-*reporter construct was diluted 1:100 into pre-warmed tryptic soy broth (TSB, Sigma Aldrich, Germany), incubated at 37 °C with agitation, and harvested at OD_600nm_ = 0.7. The bacterial cells were pelleted, washed in phosphate saline buffer (PBS, pH 7.5), and diluted in DMEM 10% FBS. Fluorescence was measured using Synergy H1 Hybrid Reader (BioTek, USA) with excitation/emission of 488/520 nm. A control sample of non-transformed *S. aureus* NCTC8325-4 was included for background correction.

### Yeast two-hybrid screen

A yeast-two hybrid service was ordered from Hybrigenics (http://www.hybrigenics-services.com/). Briefly, GAL4-SdrD-A-region was used as bait in a human reconstituted skin library.

### *S. aureus* NCTC8325-4 and NCTC8325-4Δ*sdrD* adherence and inhibition of binding to immobilized recombinant Dsg1

Adherence of *S. aureus* NCTC8325-4 and *S. aureus* NCTC8325-4Δ*sdrD* to immobilized recombinant Dsg1 was principally evaluated as described previously^39^. Briefly, microtiter plates (Nunc, Denmark) were coated with coupling buffer (100 mM sodium carbonate (Sigma Aldrich, Germany), pH 9.6) containing 1 μg/well recombinant human Dsg1-Fc chimera (R&D systems, USA) or Recombinant human IgG_1_ Fc (R&D systems, USA) and incubated overnight at 4 °C. Wells were blocked with 0.05% (w/v) bovine serum albumin (BSA) (Sigma Aldrich, Germany) for 2 h at 37 °C and washed with PBS supplemented with 0.05% (v/v) Tween (PBST). *S. aureus* NCTC8325-4 and NCTC8325-4Δ*sdrD* were grown overnight in TSB medium. The next day, bacteria were diluted and grown to an OD_600nm_ = 0.7–0.8 in TSB, washed in PBS, and suspended in PBS at OD_600nm_ = 0.8–1. Thereafter, 100 μl of bacterial suspension was added to the Dsg1-coated or IgG_1_ Fc coated wells. The plates were incubated for 2 h at 37 °C, washed with PBST, and bound cells were fixed with paraformaldehyde (4% w/v) (Sigma Aldrich, Germany) for 20–30 min and stained with crystal violet (0.5% v/v, 100 μl per well) for 1 min followed by PBS and acetic acid (5% v/v) washes. The absorbance was measured at 570 nm in an ELISA plate reader (VERSA_max_, USA). Inhibition of *S. aureus* binding to immobilized recombinant Dsg1 was evaluated as described previously^39^ by preincubating NCTC8325-4 and NCTC8325-4Δ*sdrD* with recombinant protein Dsg1-Fc (10 μg/ml), IgG_1_ Fc (10 μg/ml) or PBS for 30 min at room temperature.

### Ligand binding assay

ELISA was used to analyse the ability of SdrD-A region to bind Dsg1 as described previously^58^ with minor modifications. Briefly, different concentrations of purified SdrD-A region were added to wells of a 96-well microtiter plate (Nunc) and the plate was incubated overnight at 4 °C. Wells were washed three times with PBS containing 0.05% Tween-20 (Sigma Aldrich, Germany), blocked with 0.05% or 1% (wt/vol) filter sterilized BSA in PBS for 2h at 37 °C. Wells were re-washed and Dsg1-Fc chimera (10 μg/ml) (R&D systems, USA) were added, and GST was used as a control. The adhered Dsg1 was determined using Dsg1_471–499_ antibodies (Abgent, USA), followed by HRP-conjugated polyclonal Swine Anti Rabbit immunoglobulin (DAKO, Denmark). The measurement of the absorbance at 450 nm was carried out in an ELISA reader (VERSA_max_, USA).

### Expression of Dsg1 on the surface of HaCaT cells

To detect the expression of Dsg1 on the surface of HaCaT cells, HaCaT cells were incubated for 40 min at 4 °C with anti-Dsg1 antibodies (R&D systems, Minneapolis, MN), which is directed against the Dsg1 extracellular domain. After washing, cells were incubated for 30 min at 4 °C with the secondary antibody (Alexa 488^®^ Donkey anti goat, Thermo scientific, UK). Flow cytometry analyses were performed using FACS Fortessa (BD Bioscience).

### Binding of recombinant SdrD protein to eukaryotic cells

HaCaT cells were detached by treating with 1 × Citric Saline solution, thereafter fixed with 4% paraformaldehyde for 15 min. After washing, cells were blocked with 10% fetal bovine serum (FBS) in PBS for 15 min at room temperature. Cells were incubated for 1 hour at 4 °C with 15 μg/ml SdrD in PBS supplemented with 5% FBS (Gibco, Life Technologies, UK). Human neutrophils were incubated on ice for 30 min at 4 °C with 14 μg/ml with full-length SdrD in RPMI/HSA or 10 ug/mg FLIPr (*S. aureus* formyl peptide receptor inhibitor, a kind gift from Prof. Jos van Strijp). Bound his-tagged SdrD and FLIPr were detected using anti-his-FITC monoclonal antibody (LS Bio Sciences, USA/Abcam, UK). Flow cytometry was performed using BD Biosciences FACS Fortessa (HaCaT) and BD Bioscience FACS Calibur (Neutrophils). The fluorescence intensity (FL) of 10,000-gated cells was measured for each sample and geometric mean FL was calculated using FlowJo software (Flowjo, Ashland, OR).

### Transfection

Human full-length Dsg1 in pLKpac was a kind gift from Dr Wahl^59^. The full-length *dsg1* was amplified by PCR using primers Full Dsg1-F and Full Dsg1-R (Table 1), digested and sub-cloned into corresponding *BamHI/HindIII* site of pcDNA3 (Invitrogen). The presence of *dsg1* gene in the vector was confirmed by sequencing. HEK293 cells were seeded in 24-well plates at the concentration of 8.0–10.0 × 10^4^ cells/well. After 24 hours, transfection with pcDNA3-Dsg1 or control plasmid pcDNA3 was carried out using METAFECTENE^®^ PRO (Biontex, USA) according to the manufacturer’s instructions. The total amount of DNA used in transfection was kept constant by adding CT-DNA (Calf thymus DNA, Invitrogen, USA). A GFP reporter plasmid (pEGFP-C2) was used for monitoring transfection efficiency in each experiment. Transfection efficiency was routinely >80% for HEK293 cells.

### Growth curves of *S. aureus* NCTC8325-4 and NCTC8325-4Δ*sdrD*

*S. aureus* NCTC8325-4 and NCTC8325-4Δ*sdrD* bacteria were grown overnight in Tryptic Soy Broth (TSB). The next day, bacteria were re-grown to OD_600nm_ = 0.6 in TSB, washed, and resuspended in TSB or DMEM supplemented with 10% FCS (DMEM-FCS) at 1 × 10^5^ CFU/ml. Bacteria dilutions were added to 100-well Bioscreen Honeycomb plates and growth was monitored by OD_600nm_ measurements every 30 min under shaking conditions using Bioscreen C MBR machine at 37 °C.

### Haemolytic activity of *S. aureus* NCTC8325-4 and NCTC8325-4Δ*sdrD*

*S. aureus* NCTC8325-4 and NCTC8325-4Δ*sdrD* were streaked on the TSA plate containing 5% sheep blood and incubated overnight at 37 °C to assess their haemolytic capacity after 18 hours. The plate was tranferred to 4 °C, and evaluated again after 48 hours.

### Adhesion assay

The adhesion assay was carried out in triplicate as previously described^32^. Briefly, HaCaT cells or HEK293 cells were seeded into 24-well plates at confluent concentration of approximately 1.3–1.5 × 10^5^ cells per well in DMEM 10% FBS. HEK293 cells were transfected with indicated plasmids. The day after, the prepared bacterial strains were added to HaCaT cells and HEK293 cells at multiplicity of infection (MOI) 250 and 10 or 50, respectively. The plates were incubated for 90 min at 37 °C in a 5% CO_2_–95% air atmosphere. Non-adhered bacterial cells were washed off, and the adhered bacteria were quantified by serial dilution plating after trypsinizing (trypsin-EDTA, Sigma Aldrich, Germany) and lysing of HaCaT cells using PBS containing 0.1% Triton X-100 (Sigma Aldrich, Germany).

### Ethical Approval

Human neutrophils were isolated from freshly drawn human blood in accordance with ethical principles of the Helsinki Declaration. The blood donors provided written informed consent, and the medical ethics committee of the University Medical Center Utrecht (Utrecht, The Netherlands) approved the used protocol.

### Statistical analysis

At least three biological replicates were done for the adhesion assays and ELISA-based experiments. The specific growth rate (*k* = ((log_10_ N − log_10_ N_0_) 2.303)/(t − t_0_)) of *S. aureus* NCTC8325-4 and NCTC8325-4Δ*sdrD* exponential phase were used to compare the growth of wild type versus isogenic mutant. Statistical analysis was carried out on the data of pooled experiments. Student’s t-test in Excel was used for determination of statistically significant differences between groups (*P* <0.05). Excel was used for generating of graphs.

## Additional Information

**How to cite this article**: Askarian, F. *et al*. The interaction between *Staphylococcus aureus* SdrD and desmoglein 1 is important for adhesion to host cells. *Sci. Rep.* **6**, 22134; doi: 10.1038/srep22134 (2016).

## Supplementary Material

Supplementary Information

## Figures and Tables

**Table 1:** Primers used in this study.

Primers	Sequence (5′- ′3) →	Use	Origin
Up For *sdrD* + attB1	GGGGACAAGTTTGTACAAAAAAGCAGGCTGACTCGGATAGCGACTCAGAC	Generating fusion construct	This work
Up Rev *sdrD*	CAAGGACCTGGGTCATATTGTATAGATTACTCCTAAT TCATC	Generating and sequencing fusion construct	This work
Down For *sdrD*	GATGAATTAGGAGTAATCTATACAATATG ACCCAGGTCCTT G	Generating and sequencing fusion construct	This work
Down Rev *sdrD* + attB2	GGGGACCACTTTGTACAAGAAAGCTGGGTCGTAGC CAACCGGAATATTG	Generating fusion construct	This work
pKOR1 For	AGCTCCAGATCCATATCCTTC	Sequencing fusion construct	This work
pKOR1 Rev	CACACAGGAAACAGCTATGAC	Sequencing fusion construct	This work
*sdrD* KO confirm For	CGGTGGATTATTCGCGGC	To confirm isogenic mutant	This work
*sdrD* KO confirm Rev	CACATTTTGAAGATATGCCGTGTTG	To confirm isogenic mutant	This work
*sdrD* Int For	CGAGTGATAAAGTTGATATGCAGC	To confirm isogenic mutant	This work
*sdrD* Int Rev	AGCCTCTGTTGATGATGGCTGTAC	To confirm isogenic mutant	This work
*sdrD*- prom For	CGCCTGCAGCCAGGTCCATGTGGCCTGGTT	Generating reporter construct	This work
*sdrD*- prom Rev	CGCGGTACCCAA ATT TTTAAATAATACAAT TGTTTTAAATACAAAAAT	Generating reporter construct	This work
*sdrD-* pMG36e For	AAAATCTAGATGAATTAGGAGTAATCTAATGCT	Generating heterologous construct	This work
*sdrD-* pMG36e Rev	TTCACTCGAGCGCCTCATATAAGTTTTATTCCGT	Generating heterologous construct	This work
Full Dsg1-F	TAGTCCAAGCTTATGGACTGGAGTTTCTTC	Generating eukaryotic expression construct of full length Dsg1	This work
Full Dsg1-R	TTGTATGGATCCCTACTTGCTATATTGCAC	Generating eukaryotic expression construct of full length Dsg1	This work
SdrD-A- F	AACTGTCAAGGATTCTTAGTAGGTACAAC	Amplification of SdrD-A region	This work
SdrD-A-R	ACGTAACGTAAGATCTTTAGGTTTGTAAATACC	Amplification of SdrD-A region	This work
SdrD- Full length-_F	ATAGCGGCCGCTGTTTCTGGTAATGCTTTTGCTTTTGCTTTATTGTGATGG	Amplification of full length SdrD	This work
SdrD- Full length- R	CGCGGATCCGCAGAAAGTACTAATAAAGAATTGAACGAA	Amplification of full length SdrD	This work
