# Supplementary material for: The interaction between Staphylococcus aureus SdrD and desmoglein 1 is important for adhesion to host cells
Source: Sci Rep. 2016 Feb 29;6:22134. doi: 10.1038/srep22134 (PMC4770587; doi:10.1038/srep22134)
Supplement: Supplementary Information [file srep22134-s1.pdf]

The interaction between *Staphylococcus aureus* SdrD and desmoglein 1 is important for adhesion to host cells.

Fatemeh Askarian[1], Clement Ajayi[1], Anne-Merethe Hanssen[1], Nina M. van Sorge[2], Ingvild Pettersen[1], Dzung B. Diep[3], Johanna U. E. Sollid[1], Mona Johannessen[1]

[1]Research group of Host-Microbe Interactions, Department of Medical Biology, Faculty of Health Sciences, UiT-The Artic University of Norway, Norway.

[2]Medical Microbiology, University Medical Center Utrecht, Utrecht 3584CX, The Netherlands.

[3]Department of Chemistry, Biotechnology and Food Science, Norwegian University of Life Science, Ås, Norway.

e-mail: mona.johannessen@uit.no

Supplementary Fig. S1. Growth curves and exoprotein profiles of *S. aureus* NCTC8325-4 and NCTC8325-4Δ*sdrD*.

a

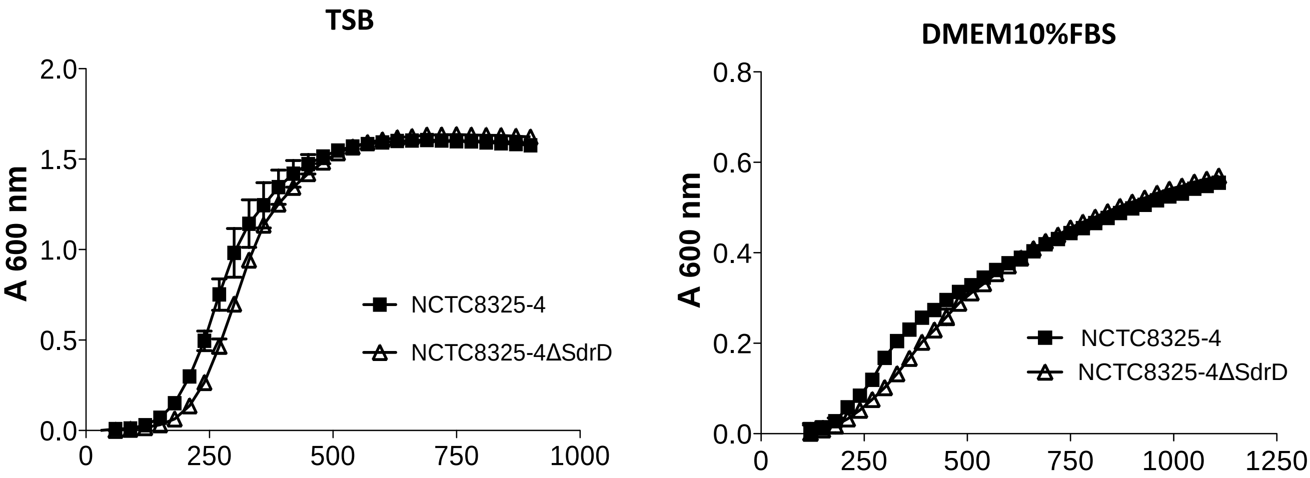

b

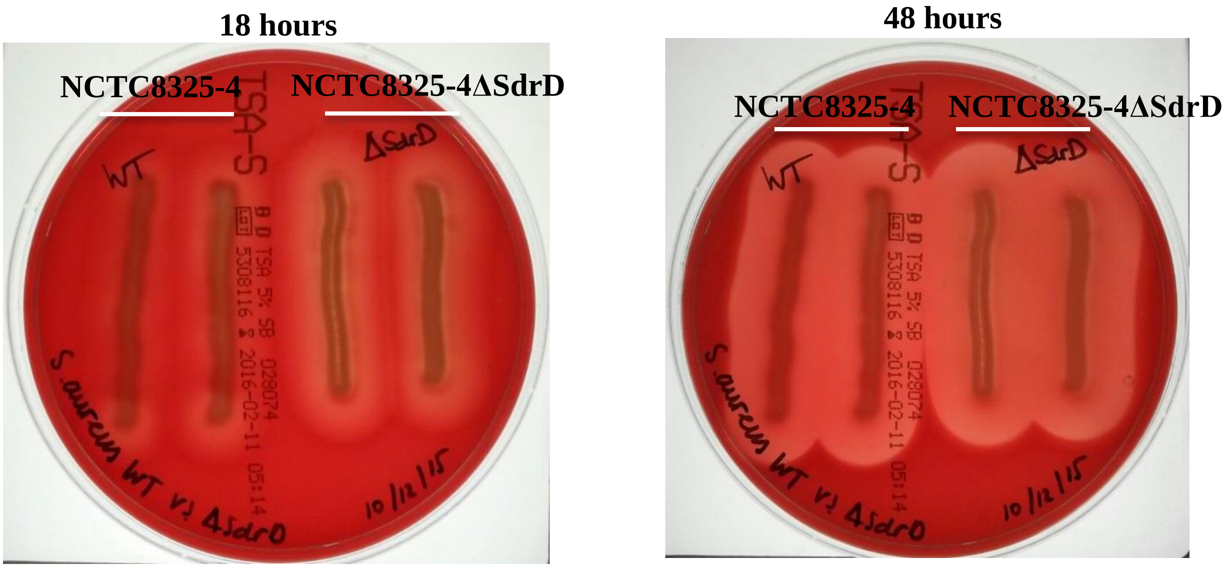

Supplementary Fig. 1. Growth curves and haemolytic activity of *S. aureus* NCTC8325-4 and NCTC8325-4Δ*sdrD*. Comparing (a) growth curves using different media and (b) haemolytic activity of wild type and isogenic mutant after 18 and 48 hours.

Supplementary Fig. S2. SdrD expression and purification.

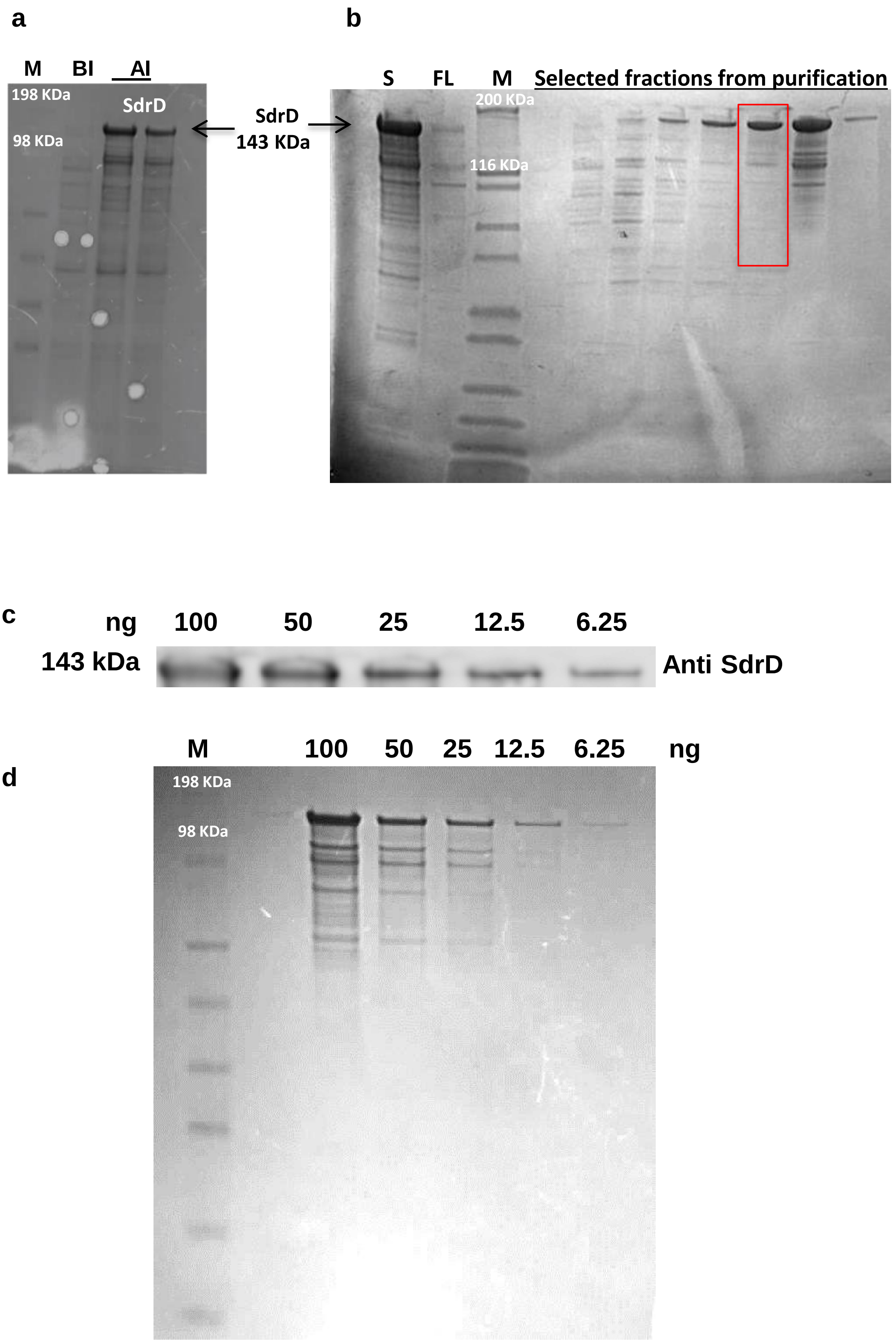

Supplementary Fig. S2. SdrD expression and purification. (a) The SdrD expression in BL21 cells before and after induction was evaluated using SDS PAGE stained by Coomassie-blue. M (See Blue-marker), BI (before induction), AI (after induction) **(b)** The SdrD was soluble and purified using HisTrap column. S (soluble fraction), FT (flow through) , M (marker, mark 12), selected fractions from the purifications. **(c)** An Immunoblot was performed on purified full-length SdrD using SdrD A-region antibodies. **(d)** Full-length SdrD was evaluated using SDS PAGE stained by Coomassie-blue. M= SeeBlue marker.

Supplementary Fig. S3 . SdrD promotes *S. aureus* adherence to the host cells expressing Dsg1.

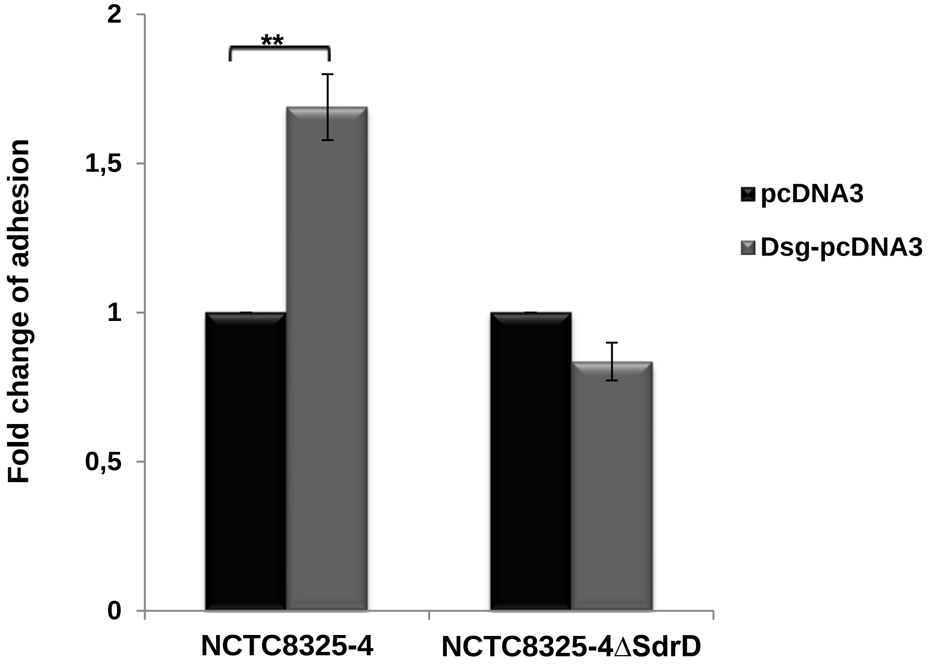

Supplementary Fig. S3. SdrD promotes adhesion to Dsg1 expressing cells. *S. aureus* NCTC8325-4 and its isogenic mutant NCTC8325-4Δ*sdrD* were added at MOI 50 to HEK293 cells transfected with pcDNA3-Dsg1 or empty pcDNA3. The cells transfected with empty vector were arbitrarily set as 1, and the adherence in pcDNA3-Dsg1 transfected is represented as fold change. Data represent means ± SEM of triplicates within a single experiments. Statistical analysis was performed by Student's t-test. Significant differences are indicated by ns (no statistical significance) or two (P<0.01) asterisks (*).
